# Antihyperthermic Treatment in the Management of Malignant Infarction of the Middle Cerebral Artery

**DOI:** 10.3390/jcm11102874

**Published:** 2022-05-19

**Authors:** Maria Luz Alonso-Alonso, Ana Sampedro-Viana, Manuel Rodríguez-Yáñez, Iria López-Dequidt, José M. Pumar, Antonio J. Mosqueira, Alberto Ouro, Paulo Ávila-Gómez, Tomás Sobrino, Francisco Campos, José Castillo, Pablo Hervella, Ramón Iglesias-Rey

**Affiliations:** 1 Neuroimaging and Biotechnology Laboratory (NOBEL), Clinical Neurosciences Research Laboratory (LINC), Health Research Institute of Santiago de Compostela (IDIS), 15706 Santiago de Compostela, Spain; anasampedro@rai.usc.es (A.S.-V.); josemanuel.pumar@usc.es (J.M.P.); drmosqueiramartinez@gmail.com (A.J.M.); jose.castillo.sanchez@sergas.es (J.C.); 2 Stroke Unit, Department of Neurology, Hospital Clínico Universitario, 15706 Santiago de Compostela, Spain; manuel.rodriguez.yanez@sergas.es (M.R.-Y.); iriaalejandralopez@googlemail.com (I.L.-D.); 3 Department of Neuroradiology, Health Research Institute of Santiago de Compostela (IDIS), Hospital Clínico Universitario, 15706 Santiago de Compostela, Spain; 4 NeuroAging Group (NEURAL), Clinical Neurosciences Research Laboratory (LINC), Health Research Institute of Santiago de Compostela (IDIS), 15706 Santiago de Compostela, Spain; alberto.ouro.villasante@sergas.es (A.O.); tomas.sobrino.moreiras@sergas.es (T.S.); 5 Translational Stroke Laboratory (TREAT), Clinical Neurosciences Research Laboratory (LINC), Health Research Institute of Santiago de Compostela (IDIS), 15706 Santiago de Compostela, Spain; pauloavilagomez@gmail.com (P.Á.-G.); francisco.campos.perez@sergas.es (F.C.)

**Keywords:** antihyperthermic treatment, ischemic stroke, leukoaraiosis, malignant infarction of the middle cerebral artery, microalbuminuria

## Abstract

Malignant infarction of the middle cerebral artery (m-MCA) is a complication of ischemic stroke. Since hyperthermia is a predictor of poor outcome, and antihyperthermic treatment is well tolerated, our main aim was to analyze whether the systemic temperature decrease within the first 24 h was associated with a better outcome. Furthermore, we studied potential biochemical and neuroimaging biomarkers. This is a retrospective observational analysis that included 119 patients. The temperature variations within the first 24 h were recorded. Biochemical laboratory parameters and neuroimaging variables were also analyzed. The temperature increase at the first 24 h (OR: 158.97; CI 95%: 7.29–3465.61; *p* < 0.001) was independently associated with a higher mortality. Moreover, antihyperthermic treatment (OR: 0.08; CI 95%: 0.02–0.38; *p* = 0.002) was significantly associated with a good outcome at 3 months. Importantly, antihyperthermic treatment was associated with higher survival at 3 months (78% vs. 50%, *p* = 0.003). Significant independently associations between the development of m-MCA and both microalbuminuria (OR: 1.01; CI 95%: 1.00–1.02; *p* = 0.005) and leukoaraiosis (OR: 3.07; CI 1.84–5.13–1.02; *p* < 0.0001) were observed. Thus, antihyperthermic treatment within the first 24 h was associated with both a better outcome and higher survival. An increased risk of developing m-MCA was associated with leukoaraiosis and an elevated level of microalbuminuria.

## 1. Introduction

The term malignant infarction of the middle cerebral artery (m-MCA) was proposed by Hacke, et al. [1] in 1996 to refer to a complication of middle cerebral artery infarction that causes high mortality (80%) and serious sequelae associated with the development of severe cerebral edema. This edema exerts a mass effect on brain structures, leading to neurological worsening. Moreover, the majority of survivors suffer from moderate to severe disabilities [2].

Current treatment options comprise medical treatment and/or decompressive hemicraniectomy. Among the former approach, only hypothermia has shown some positive results [3]. As to the latter, surgical procedures has shown improved outcomes compared with the medical treatment [4,5]. Thus, treatment of decompressive hemicraniectomy and hypothermia, alone or in combination, have been recommended during last decades. Although some studies have described the beneficial effects of these therapies [6], recent clinical trials have found a negative association with the outcome of patients [7,8]. Conversely, hypothermia alone produces more complications than benefits [9,10], and the benefit of hemicraniectomy is restricted to patients below 60 years [11,12,13,14].

We hypothesize that, since antihyperthermic treatment is well tolerated and easy to administer [15], and as hypothermia does not lead to benefits in m-MCA, avoiding hyperthermia, which is common in patients with extensive cerebral infarcts, could be associated with both a clinical benefit and a better outcome in these patients.

Therefore, the purpose of this study was to analyze whether, in patients with m-MCA, the decrease in systemic temperature in the first 24 h (with or without antihyperthermic treatment) was associated with a better outcome in an unselected sample of patients with ischemic stroke prospectively registered in a database.

## 2. Materials and Methods

### 2.1. Patient Screening

This is a retrospective observational analysis of patients with ischemic stroke admitted to the Stroke Unit of the Hospital Clínico Universitario of Santiago de Compostela (Spain), who were prospectively registered in an approved data bank, Biobanco Ictus del Complejo Hospitalario Universitario de Santiago (BICHUS). All patients were treated by expert neurologists according to national and international guidelines. Inclusion criteria for this analysis were: (1) authorization for the anonymous use of individuals’ data for research purposes; (2) neuroimaging study (magnetic resonance imaging (MRI) or computed tomography (CT) study at admission and between the 4th–7th day of hospitalization); (3) temperature control; (4) follow-up at 3 months; (5) Oxfordshire Community Stroke Project (OCSP) registration; (6) total anterior circulation infarct (TACI); and (7) m-MCA.

### 2.2. Standard Protocol Approvals, Registrations, and Patients Consents

This research was conducted in accordance with the Declaration of Helsinki of the World Medical Association (2008) and approved by the Ethics Committee of Santiago de Compostela: (2019/616). Data analysis for this study was retrospective and ran from January 2008 to December 2017. Before the start of the study, written informed consent from all participants was obtained.

### 2.3. Clinical Variables

The OCSP criteria were used for the diagnostic of a massive cerebral TACI: “acute stroke with the combination of new higher cerebral dysfunction, homonymous visual field deficit and ipsilateral motor and/or sensory deficit of at least two areas of the face, arm, and leg. If the conscious level was impaired and formal testing of higher cerebral function or visual fields was not possible, a deficit was assumed to be present” [16]. We used the following criteria described by Schwab, et al. [14] as diagnostic criteria for m-MCA: clinical evidence of acute, massive MCA infarction confirmed by CT, complete space-occupying MCA infarction with midline shift, and compression of the basal cisterns observed on follow-up CT and further neurological deterioration (reduction in the level of consciousness to somnolence or stupor with the baseline clinical status on admission). The severity of the stroke was quantified by a qualified neurologist using the NIHSS scale [17] on admission, every 6 h during the first day (or more depending on the clinical situation of the patient), 48 h, and 7 days after admission. Early neurological deterioration was defined as an increase ≥ 4 points in the NISHH scale during the first 48 h from stroke onset. Modified Rankin scale (mRs) [18] was used to evaluate functional outcome at 3 months. Stroke etiology was classified according to TOAST criteria [19]. In this study, latency time was defined as the time from the onset of symptoms to hospital admission.

### 2.4. Neuroimaging Studies

**Figure 1:**
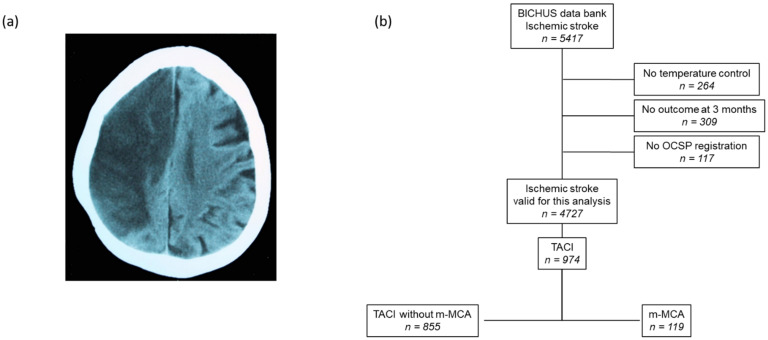
(**a**) Computed tomography scan of a patient with malignant infarction of the middle cerebral artery (m-MCA). (**b**) Flowchart of patient screening.

Neuroimaging studies included a cerebral CT scan at admission and a second CT performed between days 4 and 7 of hospitalization or earlier if there was neurological deterioration. This second CT was used to estimate the volume of the infarct (Figure 1a). In patients who were candidates for reperfusion treatment, an MRI study was carried out upon admission. Lesion volumes were calculated using an ABC/2 method [20] until 2016 and through an automated planimetric method afterward.

When available, an MRI study was carried out for the assessment of leukoaraiosis (LA). In the remainder patients, a CT scan was used for LA assessment. The presence and severity of LA was assessed according to the Fazecas’s scale in both cases [21,22]. Expert neuroradiologists blinded to clinical data performed neuroimaging evaluations.

### 2.5. Temperature Control

The patient’s axillary temperature was measured every 6 h by the nursing staff of the stroke unit. Following the stroke unit protocol, all patients with temperature ≥ 37.5 °C were treated with 1 g of paracetamol orally or 2 g of metamizole intravenously every 8 h. The treatments were maintained for at least 48 h regardless of the recorded temperature. Patients were not subjected to other hypothermic procedures.

For this analysis, the temperature on admission to the stroke unit and the highest temperature in the first 24 h were used. We considered a positive response to antihyperthermic treatment when the maximum temperature in the first 24 h was lower than the temperature on admission.

### 2.6. Treatment of m-MAC

All patients were treated by neurologists with special training in cerebrovascular diseases. Reperfusion treatment was administered on admission to patients meeting clinical and neuroimaging criteria. Decompressive hemicraniectomy was carried out in patients with m-MAC following the criteria of the neurologist and neurosurgeon on duty, in accordance with the criteria of the Spanish Society of Neurology: (1) age ≤ 60 years; (2) onset of symptoms ≤ 48 h; (3) TACI and NIHSS > 15 at time of admission; (4) early neurological deterioration; (5) infarct volume ≥ 145 cm^3^ measured by CT; and (6) stable hemodynamic status [23].

### 2.7. Outcome Endpoints

The main endpoint of the study was the association between the temperature decrease in the first 24 h and the clinical outcome (mortality and morbidity (mRS score 3–5)) at 3 months. The secondary endpoints were the influence of the antihyperthermic treatment and its repercussion on the outcome at 3 months and the existence of markers associated with the development of m-MCA, especially those related to the alteration of the permeability of the blood–brain barrier (BBB).

### 2.8. Statistical Analyses

For the descriptive study, categorical variables were described with frequency and percentage. Quantitative variables were described with the mean ± standard deviation or median and interquartile range according to the type of distribution measured by the Kolmogorov–Smirnov test for a sample with the significance correction of Lilliefors. The significance of the differences was estimated using the chi-square test, Student’s *t*-test, or Mann–Whitney U test according to the nature of the contrast variable and its adjustment to normality. Analysis of variance (ANOVA) was used to compare differences between more than two groups. Logistic regression analyses were performed to identify those variables independently associated with mortality at 3 months and development of m-MCA. The results were expressed as odds ratio (OR) with 95% confident intervals (95% CI). A *p*-value < 0.05 was considered statistically significant in all analyses. All statistical analyses were performed with SPSS V.21.0 (IBM, New York, NY, USA).

## 3. Results

### 3.1. Sample Description

A total of 5417 patients with ischemic stroke were registered in the BICHUS data bank. Of these, 690 individuals were excluded for missed temperature data, 3-month follow-up, and OCSP registration. Of the 974 patients with TACI according to the OCSP criteria, those with m-MAC (*n* = 119; 42% males vs. 58% females; mean age 64.5 ± 12.7 years) were enrolled for this analysis. The flowchart for patient screening is shown in Figure 1b. These patients were classified according to the TOAST criteria as atherothrombotic (32.8%), cardioembolic (44.5%), and indeterminate (22.7%). The body temperate at admission was 37.4 ± 0.7 °C and increased to 38.1 ± 0.9 °C in the first 24 h, which means a rise of 0.7 ± 0.8 °C during this period. Antihyperthermic treatment was performed in 59.7% of patients. The 26.1% of m-MCA individuals underwent a decompressive hemicraniectomy. Early neurological deterioration was observed in 43.3% of the subjects.

The bivariate analysis of demographic, clinical, and neuroimaging variables obtained from the patients with m-MCA at 3 months (Table 1) showed that mortality was significantly higher in females (69.2% vs. 36.6%; *p* = 0.001), patients with higher NIHSS scores at admission (*p* = 0.006), and in those patients who showed early neurological deterioration (41.5% vs. 22.0%; *p* = 0.045). The infarct volume was also a variable significantly related with mortality (222.0 ± 97.3 mL vs. 179.8 mL ± 101.1; *p* = 0.036). In contrast, mortality was lower in smokers (9.0% vs. 34.1%; *p* = 0.002), in patients undergoing thrombectomy (2.6% vs. 17.1%; *p* = 0.004), and antihyperthermic treatment (50.0% vs. 78.0%; *p* = 0.003). There were also significant differences in mortality according TOAST criteria (Table 1).

### 3.2. Association between Temperature and Functional Outcome at 3 Months

**Figure 2:**
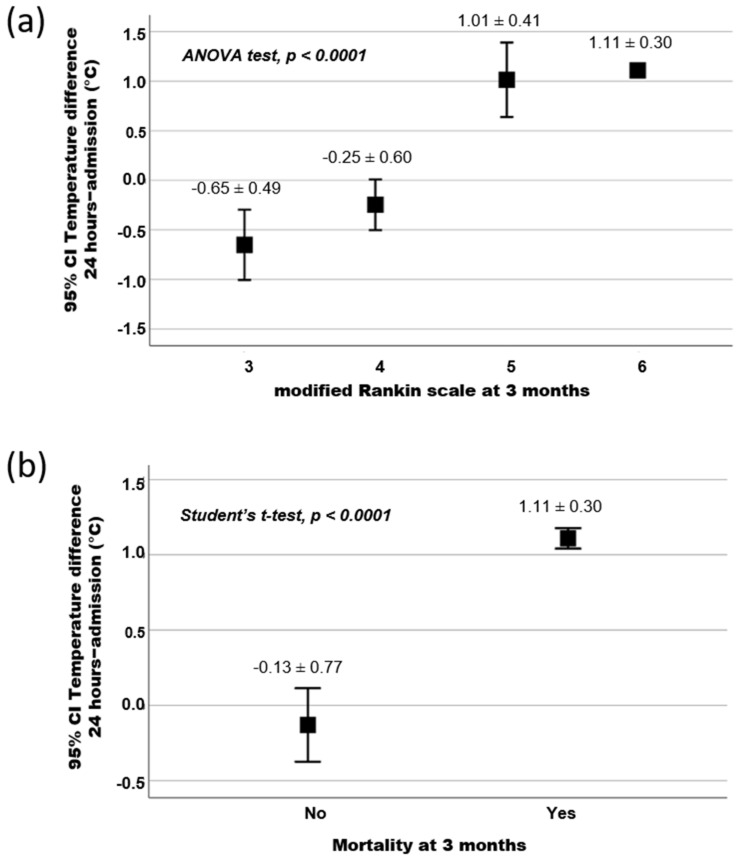
Relationship between the temperature difference in the first 24 h and (**a**) modified Rankin scale and (**b**) mortality at 3 months. (Squares represent the mean ± standard deviation).

A higher temperature (38.5 ± 0.7 °C) within the first 24 h was associated with mortality (*p* < 0.0001). In this regard, the temperature increase during this period was associated with a tendency to a poor outcome at 3 months. Furthermore, Figure 2 shows a significant association between the temperature increment in the first 24 h with morbidity, defined as a mRS score of 5–6 (*p* < 0.0001), and mortality (mRS = 6) at 3 months (*p* < 0.0001; Table 1). Moreover, the logistic regression model showed that a temperature increment during the first 24 h was independently associated (OR: 158.97; CI 95%: 7.29–3465.61; *p* = 0.001) to a higher rate of mortality in ischemic stroke patients with m-MCA (Table 2).

### 3.3. Influence of the Antihyperthermic Treatment and Its Repercussion on the Functional Outcome at 3 Months

The results of a second logistic regression model (Table 2) showed that the variables of gender (being female; OR: 5.04; CI 95%: 1.23–20.52; *p* = 0.024) and early neurological deterioration (OR: 11.47; CI 95%: 2.21–59.46; *p* = 0.004) were independently associated to poor functional outcome at 3 months. Nevertheless, antihyperthermic treatment (OR: 0.08; CI 95%: 0.02–0.38; *p* = 0.002) was independently associated with good functional outcome at 3 months. Our results show that there is a correlation between antihyperthermic treatment and better survival rates. In this regard, antihyperthermic treatment was associated with higher survival rates at 3 months (78% vs. 50%, (*p* = 0.003)). However, the influence of this treatment on the temperature differs between the data obtained at 24 h and at admission depending on the level of disability measured by the mRs. On one hand, the decrease in temperature was higher in patients with a mRS score of 3 and 4 than in those who did not receive this treatment. Moreover, this difference was significant in mRS = 4 group (*p* = 0.019). On the other hand, the increase in temperature in patients with a mRS score of 5 and 6 was lower than in those who did not receive this treatment.

### 3.4. Biomarkers Associated with Development of m-MCA

Table 3 shows a bivariate analysis used to evaluate the differences between TACI patients with and without m-MCA. Demographic variables showed statistical differences in patient age (64.5 ± 12.7 years vs. 70.1 ± 13.5 years, *p* < 0.0001) and sex (being female: 58.0% vs. 38.8%, *p* < 0.0001). The m-MCA patients exhibited a longer latency time (253.3 ± 190.1 min vs. 232.9 ± 154.2 min, *p* = 0.003), higher percentage of arterial hypertension (73.9% vs. 63.6%, *p* = 0.016), atrial fibrillation (24.4% vs. 13.1%, *p* = 0.002), and early neurological deterioration (43.3% vs. 6.8%, *p* < 0.0001). The m-MCA patient group had higher scores in both NISHSS (21 (18, 23) vs. 13 (9, 18)) at admission and mRS (6 (4–6) vs. 3 (1–4)) at 3 months (*p* < 0.0001 for both scales). When analyzing temperature, it was not only higher at admission (37.4 ± 0.7 °C vs. 36.5 ± 0.6 °C, *p* < 0.0001) but also at 24 h (38.1 ± 0.9 °C vs. 36.5 ± 1.4 °C, *p* < 0.0001) despite the antihyperthermic treatment (59.7% vs. 7.1%, *p* < 0.0001) in individuals who suffered from m-MCA. As for the molecular variables analyzed in this study, blood glucose (155.9 ± 82.4 mg/dL vs. 142.4 ± 61.6 mg/dL, *p* = 0.015), sedimentation rate (31.0 ± 17.4 mm vs. 27.9 ± 21.2 mm, *p* = 0.012), and microalbuminuria (16.3 ± 12.3 mg/24 h vs. 4.9 ± 23.7 mg/24 h, *p* < 0.0001) were significantly increased in m-MCA patients, while their 25-Hydroxy-vitamin D levels (14.7 ± 4.3 ng/mL vs. 15.2 ± 7.9 ng/mL, *p* = 0.005) were significantly lower.

Interestingly, when the value of microalbuminuria was compared in these two groups according to mRS at 3 months, its increase in m-MCA patients was confirmed. This increase was considered significant when mRS = 4 and 6 (20.2 ± 11.9 mg/24 h vs. 3.8 ± 12.4 mg/24 h and 14.8 ± 9.4 mg/24 h vs. 3.3 ± 9.6 mg/24 h, respectively; *p* < 0.0001) at 3 months. Besides, the logistic regression model for demographic and clinical variables, including microalbuminuria (Table 4), showed a significant association between this variable and the presence of m-MCA (OR: 1.01; CI 95%: 1.00–1.03; *p* = 0.005). These results suggest that microalbuminuria could be a potential marker associated with the development of m-MCA.

**Figure 3:**
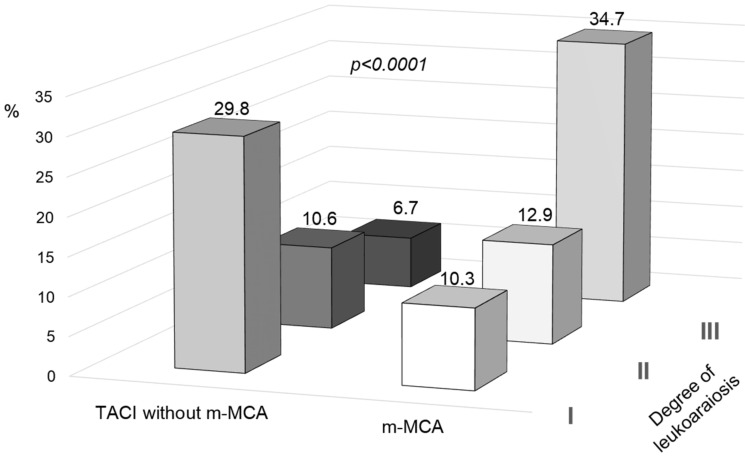
Degree of leukoaraiosis in patients with infarct in the anterior cerebral artery territory (TACI) with and without malignant infarction of the middle cerebral artery (m-MCA).

A neuroimaging variable, LA, was observed in 17.8% of patients who suffered from TACI without m-MCA, whereas this percentage increased to 58.8% in those who developed m-MCA (*p* < 0.0001). When the degree of LA was included in the analysis, degree III was seen to be predominant in the presence of m-MCA, while degree I was more prevalent (*p* < 0.0001; Figure 3 and Table 3) in patients without m-MCA. When analyzing this variable according to mRS at 3 months, the predominance of degree III of LA was observed in mRS ≥ 4 but not in mRS = 3. As observed for microalbuminuria, the logistic regression model for demographic, clinical, and neuroimaging variables, (Table 5) showed a significant association between all variables analyzed and the development of m-MCA, including LA (OR: 3.07; CI 1.84–5.13; *p* < 0.0001). This significant association was conserved when the three degrees of LA were introduced into the logistic regression model. These results support its relevance as a marker of this stroke complication.

## 4. Discussion

In 2019, stroke was the second-leading cause of death worldwide and the third-leading cause of death and disability combined [24]. The m-MCA is a complication that, although it only represent approximately 5% of all ischemic strokes, exhibits a percentage of mortality as high as 80%. Furthermore, it leads to moderate to severe disability in surviving patients, with subsequent impact on their quality of life [1,2,9,15]. Currently, the recommended treatment options are decompressive hemicraniectomy, therapeutic hypothermia, or a combination of both [4,5,6,7,8,9,11,12,13,14]. However, the risk-benefit ratio is very low, and there are several factors to take into account to decide which treatment is the most appropriate for each patient.

Hypothermia strategy in patients with ischemic brain injury is controversial [9,10,15]. It was described that an increased body temperature has deleterious effects on the ischemic brain due to an aggravation of its damage and compromising the outcome of patients [10]. In this regard, the development of hyperthermia at 24 h has been previously associated with failed reperfusion and poor clinical outcome in patients with ischemic stroke [25]. Similar to the development of cerebral edema, a hallmark symptom of m-MCA syndrome, high temperature at admission was considered a predictor [26]. Although further studies are required to confirm it, hypothermia could be a potential approach for controlling cerebral edema [15]. Therefore, our results demonstrated a slight but significant benefit in the outcome of patients with m-MCA associated with the decrease in body temperature in the first 24 h. The 3-month follow-up in these patients showed lower disability and mortality rates than those patients who experienced an increase in body temperature in the first 24 h.

The antihyperthermic treatment is feasible, well-tolerated, and easy to administer for controlling cerebral injury and edema in m-MCA patients [15]. In this study, 59.7% (71 out of 119) of all patients underwent antihyperthermic treatment. This therapy option showed a mild but significant reduction in the mortality rate at 3 months. Thus, 78% (32 out of 41) of patients who overcame the m-MCA had received antihyperthermic treatment, while this percentage was reduced to 50% (39 out of 78) in the patients who died. Our results suggest that an antihyperthermic treatment has a greater benefit than that associated with a decrease in temperature. Similar outcomes have been described in patients with intracerebral hemorrhage [27]. In this case, the decrease in body temperature in the first 24 h resulting from the administration of antihyperthermic treatment led to a better outcome at 3 months in one-third of patients [27]. However, this therapeutic treatment alone or in combination with decompressive hemicraniectomy is still controversial [6,7,8,9]. When comparing the results of these approaches should take into account not only demographic data and clinical variables but also the hypothermia protocol used, including duration, target temperature, and timing of administration.

In addition, the BBB disruption and an increase in its permeability are also key events in the pathogeny of ischemic stroke [28]. The assessment of BBB dysfunction could therefore lead to the development of new therapeutic strategies focused on this target and could also prove useful as prognostic and decision tools [29]. In this sense, high plasma levels of cellular fibronectin, a marker of BBB disruption, were associated with m-MCA development [30]. Since microalbuminuria and leukoaraiois (LA) with are described as involved in BBB disruption, we studied the potential association of these biomarkers in m-MCA [31,32,33,34,35].

Microalbuminuria is considered a notable risk-factor for cerebrovascular diseases [36,37,38]. Microalbuminuria has been widely related to the severity of ischemic stroke, hemorrhagic transformation, and poor clinical outcomes [31,36,37,38,39,40,41,42,43]. In the present study, we demonstrated an increased risk of developing m-MCA in patients with elevated levels of microalbuminuria. Likewise, our data showed that the risk of m-MCA syndrome was associated with the presence of LA in direct relation to its severity. Our data support the significant relationship recently described by Wu, et al. [44] between m-MCA and the cerebral white matter changes, which are consequently related to BBB disruption [44]. Hence, these markers could be predictors of poor outcome in these patients and a potential therapeutic target to prevent the risk of developing an m-MCA syndrome.

Nonetheless, our study is limited by its retrospective design. In addition, it is a single-center study without preclinical data to confirm the ischemic stroke as the original cause of hyperthermia. The strengths of this work are the unbiased selection of patients, the high inclusion rate following selection criteria, and the blinded analysis of data.

## 5. Conclusions

In conclusion, our study showed that the decrease of body temperature at 24 h was associated with a better outcome in patients with m-MCA. The benefits of the antihyperthermic treatment were not limited to those associated with a decrease in temperature. Furthermore, the markers of BBB disruption, such as microalbuminuria and LA, could be not only useful for prognosis and decision making but also as new targets for novel therapeutic approaches. However, further studies should be addressed to confirm our data.

## Figures and Tables

**Table 1:** Bivariate analysis of demographic data and clinical and neuroimaging variables, in the patients with m-MCA according to mortality.

Mortality	NO *n* = 41	YES *n* = 78	*p*
Age, years	60.7 ± 12.2	65.6 ± 12.6	0.080
Female gender, %	36.6	69.2	0.001
Latency time, min	252.7 ± 165.1	309.6 ± 199.8	0.157
Wake-up stroke, %	12.2	14.1	0.772
Previous TIA, %	7.3	6.4	0.851
Arterial hypertension, %	63.4	79.5	0.058
Diabetes, %	24.4	28.2	0.656
Smoking, %	34.1	9.0	0.002
Alcohol consumption, %	31.7	25.6	0.071
Dyslipidemia, %	36.6	42.3	0.545
Atrial fibrillation, %	14.6	29.5	0.073
NIHSS at admission	18 (16, 21)	21 (19, 23)	0.006
Temperature at admission, °C	37.6 ± 0.6	37.3 ± 0.8	0.134
Blood glucose, mg/dL	169.3 ± 87.9	146.8 ± 81.7	0.206
Leukocytes, ×10^3^/mL	10.7 ± 3.7	8.8 ± 0.9	0.695
Fibrinogen, mg/dL	459.6 ± 55.7	428.8 ± 69.1	0.244
C-reactive protein, mg/L	3.9 ± 3.7	3.9 ± 2.2	0.101
Erythrocyte sedimentation rate, mm	30.6 ± 15.4	31.6 ± 21.8	0.587
Microalbuminuria, mg/24 h	3.9 ± 10.4	11.6 ± 21.9	0.292
25-Hydroxy-vitamin D levels, ng/mL	14.4 ± 5.5	15.2 ± 2.3	0.659
TOAST			<0.0001
Atherothrombotic, %	36.6	30.8	
Cardioembolic, %	17.1	59.0	
Indeterminate, %	46.3	10.3	
Antihyperthermic treatment, %	78.0	50.0	0.003
Systemic thrombolysis, %	24.4	26.9	0.765
Thrombectomy, %	17.1	2.6	0.004
Hemicraniectomy, %	14.6	32.1	0.066
Leukoaraiosis, %	61.0	57.7	0.729
Degree of leukoaraiosis			0.114
Grade I, %	19.5	5.3	
Grade II, %	9.8	14.7	
Grade III, %	31.7	36.0	
Infarct volume, mL	179.8 ± 101.1	222.0 ± 97.3	0.036
Temperature 24 h	37.4 ± 0.9	38.5 ± 0.7	<0.0001
Temperature 24 h-admission	−0.14 ± 0.77	1.12 ± 0.29	<0.0001
Early neurological deterioration, %	22.0	41.5	0.045

TIA, transient ischemic attack; NIHSS, National Institute of Health Stroke Scale; TOAST, Trial of Org 10172 in Acute Stroke Treatment.

**Table 2:** Logistic regression model for demographic and clinical variables, including temperature difference 24 h admission (top) and antihypertensive treatment (bottom). Dependent variable: 3-month mortality.

	Not Adjusted			Adjusted		
	OR	CI 95%	*p*	OR	CI 95%	*p*
Female gender	3.90	1.76–8.65	0.001	6.23	0.93–41.81	0.060
Smoking	0.19	0.07–0.52	0.001	0.69	0.07–6.52	0.746
NIHSS at admission	1.12	1.01–1.24	0.026	1.08	0.87–1.35	0.493
Cardioembolic	4.11	1.47–11.43	0.007	1.39	0.21–9.27	0.730
Thrombectomy	0.13	0.02–0.65	0.013	0.74	0.01–54.79	0.891
Temperature 24 h admission	187.62	20.49–1718.39	<0.0001	158.97	7.29–3465.61	0.001
Infarct volume	1.01	1.00–1.02	0.039	1.00	0.99–1.01	0.678
Early neurological deterioration	2.52	1.01–6.33	0.048	4.33	0.67–27.79	0.123
	**Not Adjusted**			**Adjusted**		
	**OR**	**CI 95%**	***p***	**OR**	**CI 95%**	***p***
Female gender	3.90	1.76–8.65	0.001	5.04	1.23–20.52	0.024
Smoking	0.19	0.07–0.52	0.001	0.29	0.06–1.57	0.150
NIHSS at admission	1.12	1.01–1.24	0.026	1.09	0.93–1.28	0.273
Cardioembolic	4.11	1.47–11.43	0.007	2.70	0.58–12.65	0.207
Thrombectomy	0.13	0.02–0.65	0.013	0.11	0.01–1.46	0.095
Antihypertensive treatment	0.28	0.12–0.66	0.004	0.08	0.02–0.38	0.002
Infarct volume	1.01	1.00–1.02	0.039	1.00	0.99–1.01	0.103
Early neurological deterioration	2.52	1.01–6.33	0.048	11.47	2.21–59.46	0.004

NIHSS, National Institute of Health Stroke Scale.

**Table 3:** Bivariate analysis of demographic data and clinical and neuroimaging variables for patients with total anterior circulation infarct (TACI) with and without malignant middle cerebral artery (m-MCA).

	m-MCA *n* = 119	TACI *n* = 855	*p*
Age, years	64.5 ± 12.7	70.1 ± 13.5	<0.0001
Female gender, %	58.0	38.8	<0.0001
Latency time, min	253.3 ± 190.1	232.9 ± 154.2	0.003
Wake-up stroke, %	13.4	8.9	0.081
Previous TIA, %	6.7	7.8	0.419
Arterial hypertension, %	73.9	63.6	0.016
Diabetes, %	26.9	25.5	0.410
Smoking, %	17.6	20.8	0.251
Alcohol consumption, %	16.8	15.6	0.404
Dyslipidemia, %	40.3	35.7	0.186
Atrial fibrillation, %	24.4	13.1	0.002
NIHSS at admission	21 (18, 23)	13 (9, 18)	<0.0001
Temperature at admission, °C	37.4 ± 0.7	36.5 ± 0.6	<0.0001
Blood glucose, mg/dL	155.9 ± 82.4	142.4 ± 61.6	0.015
Leukocytes, ×10^3^/mL	9.9 ± 3.0	9.7 ± 3.3	0.052
Fibrinogen, mg/dL	446.7 ± 60.6	451.3 ± 94.4	0.058
C-reactive protein, mg/L	3.9 ± 3.1	4.1 ± 3.8	0.102
Erythrocyte sedimentation rate, mm	31.0 ± 17.4	27.9 ± 21.2	0.012
Microalbuminuria, mg/24 h	16.3 ± 12.3	4.9 ± 23.7	<0.0001
25-Hydroxy-vitamin D levels, ng/mL	14.7 ± 4.3	15.2 ± 7.9	0.005
TOAST			0.001
Atherothrombotic, %	32.8	43.6	
Cardioembolic, %	44.5	28.4	
Indeterminate, %	22.7	27.6	
Other, %	-	0.4	
Antihyperthermic treatment, %	59.7	7.1	<0.0001
Systemic thrombolysis, %	26.1	30.8	0.171
Thrombectomy, %	7.6	4.6	0.120
Hemicraniectomy, %	26.1	-	
Leukoaraiosis, %	58.8	17.8	<0.0001
Degree of leukoaraiosis			
Grade I, %	10.3	29.8	
Grade II, %	12.9	10.6	
Grade III, %	34.7	6.7	
Infarct volume, mL	205.7 ± 100.4	47.4 ± 72.8	<0.0001
Temperature 24 h	38.1 ± 0.9	36.5 ± 1.4	<0.0001
Temperature 24 h-admission	0.7 ± 0.8	0.3 ± 1.3	<0.0001
Early neurological deterioration, %	43.3	6.8	<0.0001
Rankin scale at 3 months	6 (4–6)	3 (1–4)	<0.0001

TIA, Transient ischemic attack; NIHSS, National Institute of Health Stroke Scale; TOAST, Trial of Org 10172 in Acute Stroke Treatment.

**Table 4:** Logistic regression model for demographic and clinical variables including microalbuminuria. Dependent variable: m-MCA.

	Not Adjusted			Adjusted		
	OR	CI 95%	*p*	OR	CI 95%	*p*
Age	0.97	0.96–0.98	<0.0001	0.94	0.91–0.97	<0.0001
Female gender	2.17	1.47–3.21	<0.0001	2.06	0.97–4.36	0.061
Latency time	1.00	1.00–1.01	0.001	1.00	1.00–1.01	0.007
Arterial hypertension	1.62	1.05–2.50	0.028	2.62	1.07–6.43	0.035
Atrial fibrillation	2.13	1.34–3.39	0.001	2.06	0.76–5.60	0.158
NIHSS at admission	1.21	1.16–1.25	<0.0001	1.28	1.18–1.38	<0.0001
Microalbuminuria	1.01	1.01–1.03	0.019	1.01	1.00–1.03	0.005
Cardioembolic	2.08	1.34–3.25	0.001	0.45	0.17–1.17	0.104

m-MCA, malignant middle cerebral artery; NIHSS, National Institute of Health Stroke Scale.

**Table 5:** Logistic regression model for demographic, clinical, and neuroimaging variables. Dependent variable: m-MCA.

	Not Adjusted			Adjusted		
	OR	CI 95%	*p*	OR	CI 95%	*p*
Age	0.97	0.96–0.98	<0.0001	0.93	0.91–0.95	<0.0001
Female gender	2.17	1.47–3.21	<0.0001	2.33	1.39–3.89	0.001
Latency time	1.00	1.00–1.01	0.001	1.00	1.00–1.01	<0.0001
Arterial hypertension	1.62	1.05–2.50	0.028	3.94	2.07–7.51	<0.0001
Atrial fibrillation	2.13	1.34–3.39	0.001	3.22	1.70–6.08	<0.0001
NIHSS at admission	1.21	1.16–1.25	<0.0001	1.21	1.15–1.27	<0.0001
Leukoaraiosis	6.61	4.41–9.91	<0.0001	3.07	1.84–5.13	<0.0001
	**Not Adjusted**			* Adjusted		
	**OR**	**CI 95%**	***p***	**OR**	**CI 95%**	***p***
Age	0.97	0.96–0.98	<0.0001	0.94	0.92–0.96	<0.0001
Female gender	2.17	1.47–3.21	<0.0001	1.90	1.09–3.32	0.024
Latency time	1.00	1.00–1.01	0.001	1.00	1.00–1.01	<0.0001
Arterial hypertension	1.62	1.05–2.50	0.028	3.42	1.73–6.75	<0.0001
Atrial fibrillation	2.13	1.34–3.39	0.001	2.59	1.30–5.18	0.007
NIHSS at admission	1.21	1.16–1.25	<0.0001	1.20	1.14–1.26	<0.0001
Degree of leukoaraiosis	6.61	4.41–9.91	<0.0001	3.07	1.84–5.13	<0.0001
Grade I	ref			ref		
Grade II	6.61	4.41–9.91	<0.0001	6.78	2.54–18.12	<0.0001
Grade III	6.61	4.41–9.91	<0.0001	13.49	6.01–30.29	<0.0001

* Adjusted by the same variables; the presence of leukoaraiosis was replaced by the three degrees of it. m-MCA, malignant middle cerebral artery; NIHSS, National Institute of Health Stroke Scale.

## Data Availability

The statistical analysis plan is available on request. The data bank is not available for legal and ethical reasons.
